# Interpolated Compressed Sensing for 2D Multiple Slice Fast MR Imaging

**DOI:** 10.1371/journal.pone.0056098

**Published:** 2013-02-08

**Authors:** Yong Pang, Xiaoliang Zhang

**Affiliations:** 1 Department of Radiology and Biomedical Imaging, University of California San Francisco, San Francisco, California, United States of America; 2 University of California, Berkeley/University of California San Francisco Joint Graduate Group in Bioengineering, Berkeley and San Francisco, California, United States of America; 3 California Institute for Quantitative Biosciences (QB3), San Francisco, California, United States of America; Glasgow University, United Kingdom

## Abstract

Sparse MRI has been introduced to reduce the acquisition time and raw data size by undersampling the *k*-space data. However, the image quality, particularly the contrast to noise ratio (CNR), decreases with the undersampling rate. In this work, we proposed an interpolated Compressed Sensing (iCS) method to further enhance the imaging speed or reduce data size without significant sacrifice of image quality and CNR for multi-slice two-dimensional sparse MR imaging in humans. This method utilizes the k-space data of the neighboring slice in the multi-slice acquisition. The missing k-space data of a highly undersampled slice are estimated by using the raw data of its neighboring slice multiplied by a weighting function generated from low resolution full k-space reference images. In-vivo MR imaging in human feet has been used to investigate the feasibility and the performance of the proposed iCS method. The results show that by using the proposed iCS reconstruction method, the average image error can be reduced and the average CNR can be improved, compared with the conventional sparse MRI reconstruction at the same undersampling rate.

## Introduction

Compressed Sensing [1], [2] technique has been applied to MRI to significantly reduce the raw data required for image reconstruction by undersampling the k-space using an incoherent sampling strategy [3]. With compressed sensing, it is possible to shorten the acquisition time or enhance the image resolution by designing specific MRI sequences, and therefore improve the quality of MR images [4]–[20] and MR spectroscopy [21]–[25]. The sampling strategy and reconstruction method are key elements to achieve high quality images from significantly undersampled k-space data in compressed sensing MRI. Over the past few years, various sampling strategies and reconstruction methods based on compressed sensing have been developed to enhance the MR image and spectroscopy quality [26]–[41].

In this work, we propose an interpolation method to further improve imaging speed or reduce raw data size while preserve the image fidelity and contrast to noise ratio (CNR) for multi-slice two-dimensional sparse MR imaging in humans. This method utilizes the k-space data from the neighboring images to compensate for the missed k-space data of the target slice. The difference of the anatomic stricture between the adjacent slices is estimated by dividing the neighboring slice images in image domain and the quotient is transformed to k-space to form a weighting function. The raw data of the neighboring slice is convolved by the weighting function and used to estimate the missed k-space data of the target slice. This treatment helps improve the image quality, and especially the image CNR. In-vivo MR imaging of human feet has been used to investigate the feasibility and the performance of the proposed method. The experimental results show increased image quality using the proposed interpolated Compressed Sensing (iCS) reconstruction over the conventional compressed sensing technique at the same undersampling rate.

## Theory and Methods

**Figure 1:**
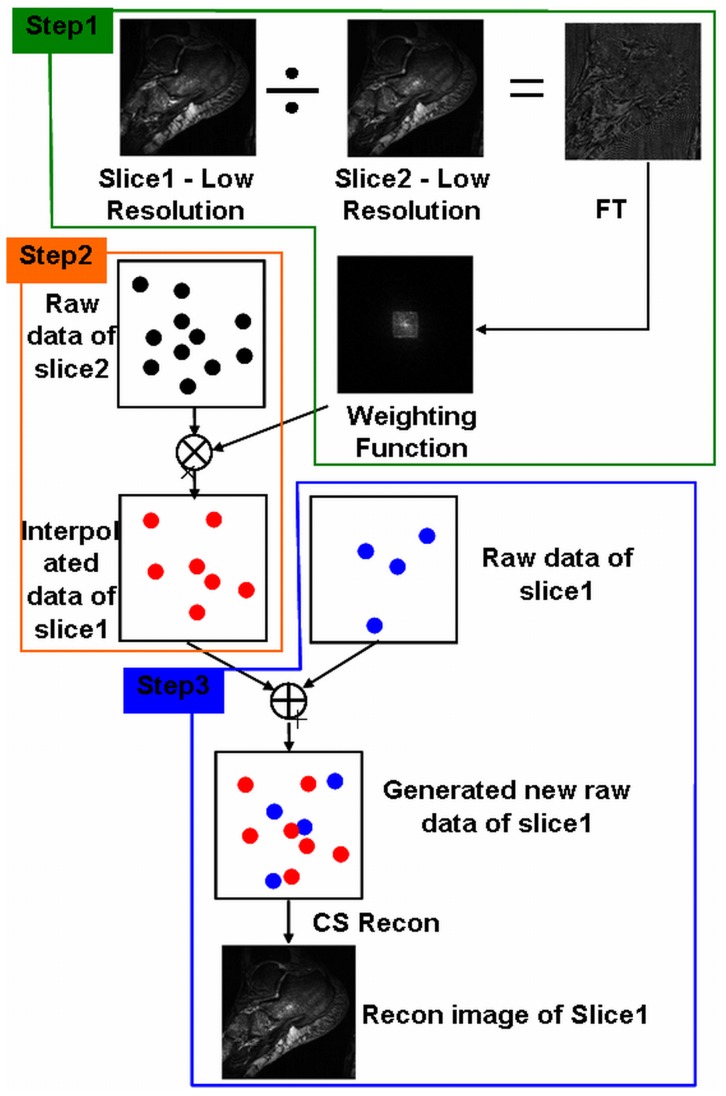
Diagram of the proposed interpolated Compressed Sensing (iCS) method. It contains total 3 steps: Step1, calculating the weighting function which maps the raw data from the neighboring slice (Slice2) to the target slice (Slice1) by acquiring low resolution full k-space images of the two adjacent slices; Step2, estimating the missed k-space data of the target slice (Slice1) by using the raw data of its neighboring slice (Slice2) convolved by the weighting function; Step3, k-space data interpolation and image reconstruction of the target slice (Slice1).

In multi-slice two-dimensional MR imaging, the raw data of two adjacent slices have structural similarity, therefore it is possible to increase the image quality by interpolating the missed k-space data of the target slice by using the k-space data from the neighboring slice and a weighting function, while keep the undersampling rate unchanged. The weighting function is estimated by taking Fourier transform of the quotient between the adjacent slices in question. Fig. 1 shows a flowchart describing the proposed method interpolated Compressed Sensing (iCS). Here for convenience, slice1 is defined as the target image with high undersampling rate (e.g., 1/100), while slice2 is defined as the image with low undersampling rate (e.g., 1/4). The raw data of slice2 was used to estimate the missed data of slice1 and interpolated them into the k-space of slice1 after convolving the weighting function.

### Estimation of the weighting function in k-space

The first step is to estimate the weighting function which is necessary to transfer the raw data of one slice to its adjacent slice. Theoretically, there exists a weighting function which maps the k-space data from one slice to another. In this work, the weighting function in image domain was first estimated by calculating the quotient between the images of the target slice and its neighboring slice, and then the weighting function in k-space domain was obtained by taking Fourier Transform of its image domain formation. Low resolution image of each slice was firstly acquired and the quotient of the two low resolution images was taken to obtain the weighting function in image domain:
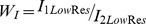
(1)where *I_1LowRes_* and *I_2LowRes_* denote the low resolution image of slice1 and slice2 respectively. By taking Fourier Transform of the *W_I_*, the weighting function in *k*-space domain was obtained:
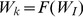
(2)where *W_k_* is the weighting function in *k*-space. In the proposed reconstruction method, the weighting function determines the accuracy of the interpolated data and the image error.

### Calculation of the interpolated raw data for slice1

To describe the reconstruction procedure in iCS multi-slice sparse MR imaging, we assume one slice (e.g., Slice1) is undersampled at a high undersampling rate while its adjacent slice (e.g., Slice2) is undersampled at low undersampling rate. The missing data of slice1 is then estimated by using the raw data of slice2 convolved by the weighting function:
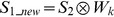
(3)where *S*_2_ is the raw data of the slice2 undersampled at a low rate (1/4), while *S*_1_new_ is the estimated raw data of slice1 which also has a low undersampling rate of 1/4.

### Missed k-space data interpolation for the highly undersampled target slice (slice1)

As we already acquired the raw data *S*_1_ of slice1 undersampled at a high undersampling rate (1/100), we kept the original raw data *S*_1_ and interpolated the missed data from *S*_1_new_. Thus an interpolated raw data of slice1 *S*_1_int_ is obtained. By taking nonlinear Conjugated Gradient (CG) reconstruction as that used in conventional compressed sensing MRI, an improved image of slice1 is then obtained.

**Figure 2:**
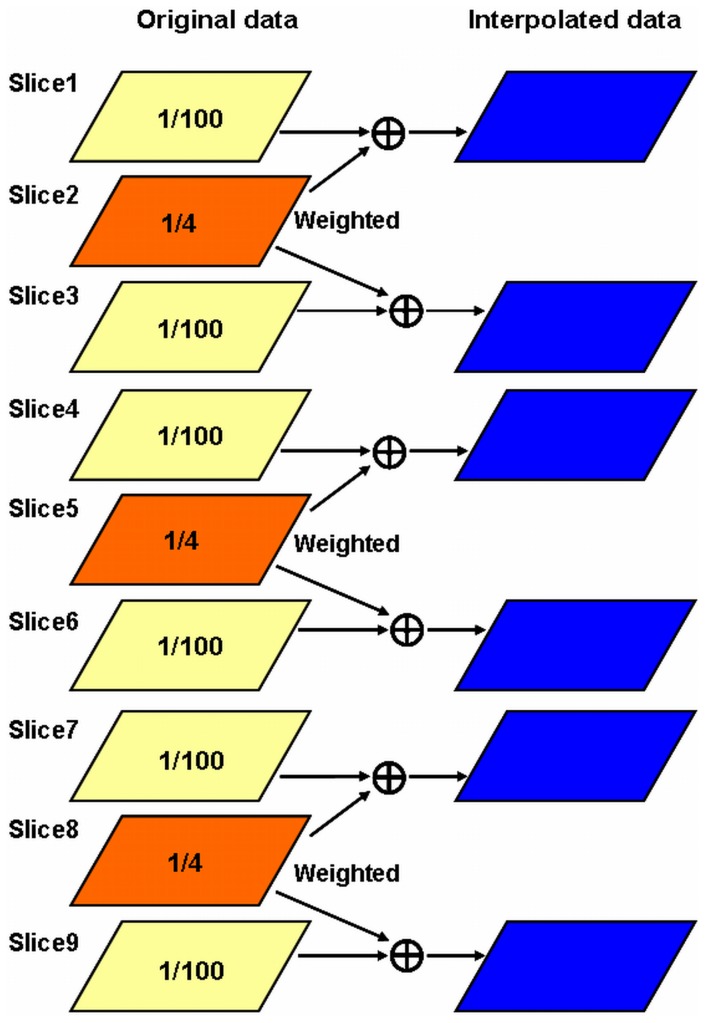
Multi-slice two dimensional sparse MR imaging strategy used in the MR experiment. Total 9 slices were obtained and were grouped into 3 groups. In each group the slice at the middle was undersampled at low undersampling rate of 1/4, while the other two slices were undersampled at rate of 1/100. The middle slice was thus used to estimate the missed k-space data of the two neighboring slices. That is, the slices of #2, #5 and #8 were undersampled at 1/4 rate and used to interpolate the missing k-space data of the slices of #1 and #3, #4 and #6, #7 and # 9, respectively.

Based on this algorithm, a multi-slice acquisition strategy to accelerate image acquisition is developed and exemplified using 9-slice 2D human foot images. As shown in Fig. 2, the slices were divided into groups and thus within each group there were 3 slices for this 9-slice 2D image example. In each group the second slice was sampled at a low undersampling rate of 1/4, while the first and the third slices were undersampled at a high undersampling rate of 1/100. The raw data of the second slice could be utilized to estimate the missing data in its neighboring two slices. In our experiment, the healthy human foot images used for investigating the proposed iCS image reconstruction were acquired using a microstrip RF coil on a whole body ultrahigh field 7T MR scanner (GE Healthcare, Milwaukee, WI) [42]–[45]. The 7T imaging study procedure was approved by the Committee on Human Research (CHR) of University of California San Francisco (UCSF). The participants provided their written informed consent to participate in this imaging study. The three slices, i.e. the 2^nd^, 5^th^ and 8^th^ slices, in this acquisition were undersampled at the rate of 1/4, while all the other slices were undersampled at the very high undersampling rate of 1/100 as shown in Fig. 2. The other imaging parameters were: Receiver bandwidth = 15.6 kHz, flip angle = 30°, TE = 2.76 ms, TR = 10 ms, matrix size = 512×512, field of view (FOV) = 14 cm, phase FOV = 1, slice thickness = 2 mm, slice spacing = 2 mm, number of excitation = 1, the phase encoding direction is Superior-Inferior, In-plane resolution was 0.276 mm.

Image errors in the undersampled images using conventional CS and the proposed iCS methods were calculated to evaluate the reconstruction performance. In this calculation, the image errors were obtained by subtracting the reconstructed images from the full k-space reference images. Specifically, the image error calculation used can be described as
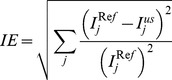
(4)where 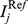 represents the signal intensity of the *j*th pixel in the full k-space reference image, and 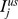 represents the signal intensity of the *j*th pixel in the undersampled image using the conventional CS or the proposed iCS methods.

To further investigate the performance of the proposed iCS strategy, we have compared the iCS multislice acquisition strategy at the mixed undersampling rates of 1/100 and 1/4 and the regular CS acquisition at equal undersampling rate of 1/11 of all slices. These two cases, i.e. iCS at undersampling rates of 1/100 and 1/4 and CS at undersampling rate of 1/11, have the same total acquisition time.

## Results

**Figure 3:**
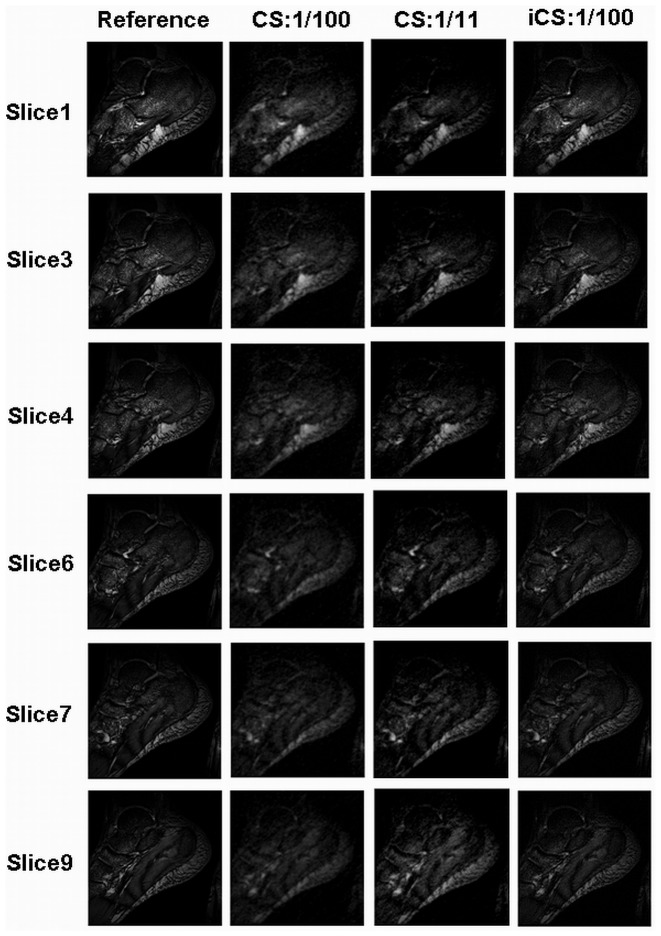
In-vivo MR images of human feet in sagittal plane. Each slice (i.e., slice #1, #3, #4, #6, #7, and #9) was reconstructed using the three methods: conventional full k-space reconstruction (which served as the reference in the 1^st^ column), conventional Compressed Sensing at undersampling rate of 1/100 (in the 2^nd^ column), conventional Compressed Sensing at undersampling rate of 1/11 (in the 3^rd^ column), and the proposed interpolated CS methods at the undersampling rate 1/100 (in the 4^th^ column). In this comparison study, image quality improvement of the iCS reconstruction method is observed compared with the CS method, much image details being recovered.

**Figure 4:**
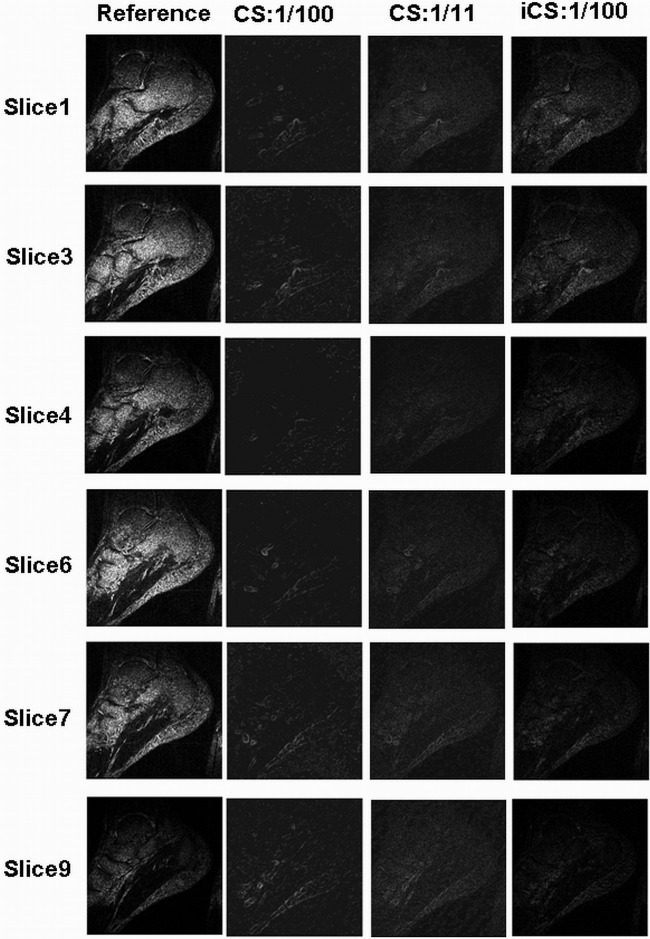
The Contrast to Noise Ratio (CNR) maps of the full k-space reference images, CS reconstructed images at 1/100 & 1/11 undersampling rates and the iCS reconstructed images shown in **Fig. 3**. It is demonstrated that the CNR is significantly improved by using the iCS method compared with the CS method at the same undersampling rate of 1/100 or the same acquisition time.

Fig. 3 shows the foot images of 6 slices on sagittal plane (the slice numbers are #1, #3, #4, #6, #7, #9). The first column is the images reconstructed from full k-space data acting as the reference; the second and third columns are the images reconstructed using the original CS at undersampling rate of 1/100 and 1/11 respectively; the fourth column is the images reconstructed using the proposed iCS method at 1/100 undersampling rate. The experiment results show that the contrast is greatly improved when using the iCS method to estimate the missed k-space data for reconstruction. The contrast to noise ratio (CNR) was calculated point by point and plotted in 2D figures as shown in Fig. 4. For slice 1, the average CNR of the reference image is 54.5, while that of the CS reconstructed image (1/100 undersampling rate), CS image (1/11 undersampling rate) and iCS reconstructed image (1/100 undersampling rate) are 5.3, 9.7 and 31.5 respectively. The results of other slices are shown in Table 1. These results show an average 4-fold CNR improvement in iCS reconstructed images over the CS reconstructed images at the high undersamling rate of 1/100, and an average 2-fold CNR improvement over CS reconstructed images with the same acquisition time.

**Table 1:** The average CNR values of the full k-space reference image, the CS reconstructed image at 1/100 & 1/11 undersampling rates and the iCS reconstructed image of each slice.

		Slice1	Slice3	Slice4	Slice6	Slice7	Slice9
Average CNR	Ref	54.5	62.3	45.5	67.4	50.9	21.6
	CS 1/100	5.3	6.3	4.1	5.0	6.9	6.6
	CS 1/11	9.7	12.6	8.9	11	12.5	13.9
	iCS	31.5	28.7	19.2	20.0	20.4	16.1

By using the iCS method, the CNR of each slice is significantly improved compared with that of the CS method at the same undersampling rate or the same acquisition time.

**Figure 5:**
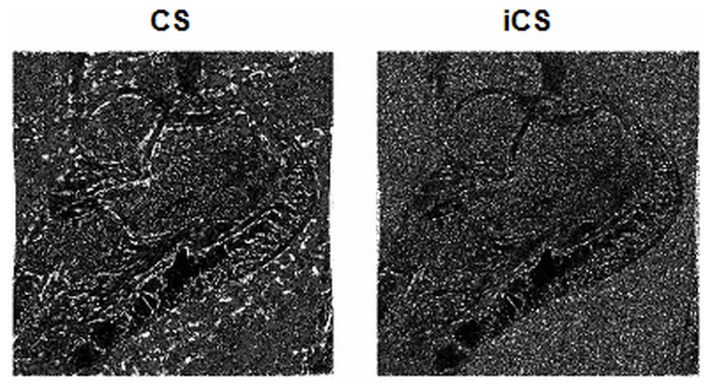
Image error maps. The image error maps are calculated by subtracting the CS constructed image (a) and the iCS constructed image (b) from the full k-space reference image. This result shows a much reduced image error in the iCS reconstructed image over the conventional CS reconstructed image.

The results of the image errors calculated by subtracting the reference full k-space images from the CS or iCS reconstructed images are shown in Table 2. This calculation is based on the images shown in Fig. 3. The average image error of the iCS constructed images was 0.0072, while that of the CS constructed images (1/100) was 0.0100, showing approximate 40% enlargement of image error in CS images comparing with the iCS images. The average image error of CS images at 1/11 undersampling rate was 0.0083, showing 15% enlargement of image error compared with the iCS images. Fig. 5 illustrates the image error maps of the CS reconstructed image and the iCS reconstructed image. In this comparison, the proposed iCS method shows a much reduced image error over the conventional CS method, demonstrating its excellent capability of preserving image fidelity at high undersampling rate.

**Table 2:** The average image error of the CS reconstructed image and the iCS reconstructed image compared with the full k-space reference image.

		Slice1	Slice3	Slice4	Slice6	Slice7	Slice9
Average Image error	CS 1/100	0.0122	0.0094	0.0098	0.0100	0.0097	0.0088
	CS 1/11	0.0108	0.0072	0.0075	0.0080	0.0078	0.0082
	iCS 1/100	0.0090	0.0066	0.0070	0.0075	0.0076	0.0052

By using the iCS method, the image error of each slice can be reduced compared with that of the CS method at the same undersampling rate or the same acquisition time, resulting in better image quality.

## Conclusions and Discussion

The interpolated Compressed Sensing reconstruction method proposed in this work provides a way to reducing acquisition time and image data size or improving the image quality, especially contrast to noise ration (CNR) for undersampled multi-slice, two-dimensional MRI. In this method, the missed k-space data of the target slice are estimated using the neighboring slice k-space data weighted by the weighting function generated from low resolution full k-space reference images of the target slice and its neighboring slice. The highly undersampled k-space data of the target slice are interpolated using these estimated data and are reconstructed based on the conventional nonlinear Conjugant Gradient method. In-vivo MR images of human feet are reconstructed with both the conventional CS and the iCS methods to demonstrate the feasibility and performance of the proposed iCS approach.

In the imaging examples demonstrated in this work, the conventional compressed sensing shows a limitation at a high undersampling rate, e.g., 1/100. The image quality was significantly degraded and the reconstructed images became blurring. The CNR of the images decreased to approximately 10% of the reference full k-space data at the same resolution, losing most structural details of the image. The proposed iCS method exhibits the capability of preserving image quality, especially the CNR, at high undersampling rate. This method allows a much faster acquisition without significant sacrifice of imaging CNR. In this method, due to the use of the image information from neighboring slice, higher image fidelity can be obtained, particularly when the gap between the two neighboring slices is small and the slice thickness is thin. When the iCS method is used in SENSE parallel imaging, the reference images required in SENSE imaging can be employed for calculating the weighting function.
